# Draft Genome Sequence of *Burkholderia* sp. MR1, a Methylarsenate-Reducing Bacterial Isolate from Florida Golf Course Soil

**DOI:** 10.1128/genomeA.00608-15

**Published:** 2015-06-04

**Authors:** Shashank S. Pawitwar, Sagar M. Utturkar, Steven D. Brown, Masafumi Yoshinaga, Barry P. Rosen

**Affiliations:** a Department of Cellular Biology and Pharmacology, Herbert Wertheim College of Medicine, Florida International University, Miami, Florida, USA; b Graduate School of Genome Science and Technology, University of Tennessee, Knoxville, Tennessee, USA; c Biosciences Division, Oak Ridge National Laboratory, Oak Ridge, Tennessee, USA

## Abstract

To elucidate the environmental organoarsenical biocycle, we isolated a soil organism, *Burkholderia* sp. MR1, which reduces relatively nontoxic pentavalent methylarsenate to the more toxic trivalent methylarsenite, with the goal of identifying the gene for the reductase. Here, we report the draft genome sequence of *Burkholderia* sp. MR1.

## GENOME ANNOUNCEMENT

Arsenic is considered to be the most prevalent environmental toxin by the U.S. Environmental Protection Agency (EPA). Prolonged exposure to arsenic in drinking water results in diseases such as skin, bladder, and prostate cancer (1–3). Pentavalent organoarsenical herbicides such as monosodium methylarsenate (MSMA) [MAs(V)] undergo environmental reduction to the more toxic trivalent species and eventual C-As bond cleavage to produce arsenite [As(III)] (4), which contaminates our drinking water supplies (5). The poultry growth promoter roxarsone (4-hydroxy-3-nitrophenylarsonic acid) has been proposed to undergo a similar pathway of reduction and C-As bond cleavage (6). The *arsI* gene of soil organisms encodes a C-As bond lyase that catalyzes the second reaction, but the pathway for reduction of MAs(V) to MAs(III) is unknown. The objective of this study was to identify the gene(s) and enzyme(s) responsible for MAs(V) reduction.

MSMA is applied as an herbicide to golf courses in Florida. We isolated an MAs(V)-reducing organism from simulated golf course soil. From the 16S rRNA gene sequence, it is a Gram-negative bacterial strain closely related to *Burkholderia glathei* (4). The environmental isolate has been named *Burkholderia* sp. MR1. Genome sequencing was performed using an Illumina HiSeq platform, with quality-based trimming, as described previously (7). After trimming, 6,727,114 paired-end reads remained, with an average read length of 90 bp, comprising genome coverage of 100×. After evaluation of several approaches (8), optimal assembly was obtained through SPAdes software (version 3.1.1). The assembly consisted of 58 large contigs (≥500 bp), with a total genome size of 6.01 Mb, an *N*_50_ contig size of 244 kb, and the largest contig of 432 kb. Gene prediction and annotation were performed at Oak Ridge National Laboratory, as described previously (9). The draft genome sequence has 5,554 candidate protein-coding genes and a G+C content of 63.2%.

The genomic sequence of *Burkholderia* sp. MR1 has a single *ars* operon comprising the genes *arsH* [encoding an NADPH-dependent flavin mononucleotide (FMN) MAs(III) oxidase] (10), *acr3* [encoding an As(III) efflux permease] (11), and *arsR* [encoding an As(III)-responsive repressor protein] (12). The operon is on the complementary strand, so the putative operon structure is *arsR-acr3-arsH*. Note that ArsH detoxifies MAs(III) (10) and so may protect this organism from the toxicity of the product of the reductase. None of these gene products catalyze MAs(V) reduction, so the gene or genes for reductase activity must be elsewhere in the chromosome. In the genome there are 195 genes annotated as reductases that are candidates for the MAs(V) reductase. However, further biochemical or genetic analysis is required to identify the responsible gene(s).

### Nucleotide sequence accession number.

The draft genome sequence of strain AE038-8 has been deposited at DDBJ/EMBL/GenBank (accession number JWHM00000000). The version described in this paper is the first version.
